# Training the Concept of Innovate in Dolphins (*Tursiops truncatus*) Is Both Creative and Cognitively Stimulating

**DOI:** 10.3390/ani14060896

**Published:** 2024-03-14

**Authors:** Deirdre B. Yeater, Kathleen M. Dudzinski, Dawn Melzer, Andrew R. Magee, Michaela Robinett, Gonzalo Guerra, Kimberly Salazar, Teri Bolton, Heather Manitzas Hill

**Affiliations:** 1 Psychology Department, Sacred Heart University, Fairfield, CT 06825, USA; yeaterd@sacredheart.edu (D.B.Y.); melzerd365@sacredheart.edu (D.M.); 2 Dolphin Communication Project, P.O. Box 7485, Port St. Lucie, FL 34985, USA; kdudzinski@dolphincommunicationproject.org; 3 Psychology Department, St. Mary’s University, San Antonio, TX 78228, USA; a.magee@tcu.edu (A.R.M.); mrobinett@mail.stmarytx.edu (M.R.); gguerra3@mail.stmarytx.edu (G.G.); ksalazar11@mail.stmarytx.edu (K.S.); 4 Roatán Institute for Marine Sciences, Anthony’s Key Resort, Sandy Bay, Roatán 31000, Honduras; t_bolton@anthonyskey.com

**Keywords:** innovate stimulus, creativity, fluency, flexibility, originality, delphinid, marine mammal

## Abstract

**Simple Summary:**

Creative or novel behaviors in bottlenose dolphins (*Tursiops truncatus*) can be indicators of flexible thinking and problem solving. Twelve bottlenose dolphins (five females, seven males) in managed care were reinforced for exhibiting different behaviors of their choosing in response to a hand gesture. Using a human-based theory of creativity, the dolphins’ behaviors were assessed for four aspects: how many different behaviors they could produce in a session or in a row (fluency), how different the behaviors were from each other (flexibility), how simple or complex the behaviors were (elaboration), and how novel or new the behaviors were (originality). The results indicated that dolphins were variable in all aspects measured, with some animals producing more behaviors that were also more complex and variable in type and energy than the other dolphins. Behaviors were also invented by several dolphins. The dolphins were engaged and cognitively challenged by this task, which suggests this task facilitates cognitive welfare while providing a means to study innovative behavior across species.

**Abstract:**

Creative or novel behaviors in bottlenose dolphins (*Tursiops truncatus*) can be indicators of flexible thinking and problem solving. Over 50 years ago, two rough-tooth dolphins demonstrated creative novel behaviors acquired through reinforcement training in human care. Since this novel training, a variety of species have been trained to respond to this conceptual cue. The current study assessed the creativity of 12 bottlenose dolphins (5 females, 7 males) housed at the Roatan Institute for Marine Sciences (RIMS) in Roatan, Honduras. Individual differences were found across four constructs measured for creativity: fluency, flexibility, elaboration, and originality. Variability in performance occurred across test sessions. Animals with less experience with this task performed fewer “innovative” behaviors as compared to more experienced animals. Despite errors, dolphins continued to attempt the task during test sessions, suggesting the concept of “innovate” was intrinsically rewarding and cognitively engaging. This task may be utilized across species to promote the comparative study of innovative or creative behavior as well as to promote cognitive welfare.

## 1. Introduction

Ecological specializations have evolved in myriad dolphin populations, including foraging innovations [1,2] and novel sounds [3,4]. Creativity may also be represented in the play behaviors of dolphins, which shows both flexibility and innovation in interactions with objects within their environments [5,6,7,8]. The variation within foraging solutions or acoustic repertoires demonstrates natural conditions or ecological problems in which innovation or creativity may manifest.

Some relevant life history parameters that possibly influenced the conditions under which innovation or creativity emerged for bottlenose dolphins (*Tursiops truncatus*) include their habitat, social structure, and developmental course. Bottlenose dolphins inhabit temperate waters around the world and live in relatively stable, complex societies with a fission–fusion social structure. Female dolphins show natal philopatry, returning to their primary ranges as adults to calve, while male dolphins appear to range throughout multiple areas [9,10,11]. Female and male dolphins may be found in mixed-sex, similar-aged social groups that travel broadly until reaching adulthood and transitioning to separate adult male social groups and female social groups with their offspring [10,11].

Bottlenose dolphins have been in human care for more than 50 years, during which many aspects of their physiology, anatomy, health, behavior, acoustics, and cognition have been studied [12,13,14]. Research has indicated that the lifespan of bottlenose dolphins in managed care is now longer, on average, than bottlenose dolphins in the wild [15]. One question that emerges when considering the life span of dolphins in managed care is what factors enhance their life spans? For a species that spends much of its life solving problems such as finding food, finding a mate, finding compatible associates, and exploring novel objects within their habitat, dolphins in managed care propagate these cognitive abilities, which evolved based on these environmental pressures. Thus, dolphins in human care require myriad types of cognitive and social stimulation and enrichment to maintain their health and overall well-being.

Some issues faced in managed care by big-brained animals that live in complex fission–fusion societies include maintaining a stable, yet socially complex, group structure that enables choice and control for companions, activities, novelty, and cognitive challenges [16]. Whether a dolphin or a dog, animals in managed care (ex situ) should have opportunities for rest but should also be cognitively stimulated using variable schedules to simulate in situ conditions [17]. Animals that have too few things to keep themselves occupied eventually invent behaviors that may be stimulating but may also have a negative impact on their welfare (e.g., regurgitation of fish that becomes an enrichment object to be manipulated or patterned behaviors that increase activity or movement but create calluses or rub marks, personal observations, H. Manitzas Hill; D. Yeater; [18]). These invented behaviors unfortunately have conflicting functions that may affect overall well-being and must be managed by providing physical, social, and cognitive stimulation that enhances the overall quality of life [19,20,21,22,23,24].

### 1.1. Ways to Enrich Managed Care Animals

Enrichment, or provisioning an animal’s habitat with supplemental stimuli, has been categorized into five different types: sensory-based, structural modifications, nutritional variation, cognitive problems, and social options [20,21]. For dolphins, social and cognitive forms of enrichment may be particularly effective for enhancing well-being, as suggested by an increased focus of empirical studies on this topic [20,23,25,26]. Social enrichment provides opportunities for animals to choose between available companions or to be autonomous. Interestingly, social enrichment can include conspecifics, other non-human companions, or humans, and it may also increase opportunities for reproduction; the presence of resulting calves is known to diversify behavioral repertoires in cetaceans [27]. Cognitive enrichment includes a wide range of options from training sessions, enrichment devices that pose problems to be solved on their own, pool configuration that creates a giant obstacle course, or other surprising or novel events [19,20,21,22,23,24].

### 1.2. Training

Training sessions include shaping and maintaining a variety of behaviors such as husbandry behaviors (e.g., blood draw, body, mouth, and eye inspections, ultrasounds), new behaviors (e.g., combinations of current behaviors or adding new behaviors to the repertoire), desensitization of novel, unusual, or startling stimuli (e.g., medical equipment, habitat structures, gates, sounds), relationship building between animals and humans and between animals, exercise (e.g., aerials, fast swims throughout the habitat, at the surface, and at depth), and different cognitive challenges (e.g., problem solving task, discrimination task, generalization tasks, human–animal interaction programs when variable in presentation) [28,29]. Each activity described provides opportunities for the animals to be cognitively engaged, demonstrate choice, and actively control the outcomes of a session (e.g., choose to cooperate with trainers and other animals, be reinforced with food or time with trainers, or choose to spend time with other animals versus training staff [12]). If variability between types of sessions and within sessions is present, cognitive enrichment increases, as the animals will have greater difficulty predicting the upcoming activities. Recent research has demonstrated the reinforcing properties of training sessions and human–animal interactions with dolphins and other marine mammals [21,26,30]. Bottlenose dolphins exhibited anticipatory behavior as training sessions were about to begin [25,31,32,33,34], showed positive affect when interacting with trainers [35], and illustrated excitement when reinforced for performing a behavior (i.e., victory squeal [36]). One trained behavior that has been noted as intrinsically reinforcing for marine mammals is the “innovate” behavior [5,34,37,38,39]. Once animals understood the concept of producing a behavior that is different (or novel) from previous behaviors, sessions became longer, and some animals diversified their exhibited behaviors [5,34,37,38]. These previous studies suggest that this concept is not only cognitively stimulating, but it also offers animals the opportunity to exert choice over the behaviors they exhibit and control over the session once the rules have been established [12,37].

### 1.3. Creativity

#### 1.3.1. Spontaneous Creativity

Within delphinids and many other aquatic and terrestrial animals, the concept of creativity is represented typically by innovations in foraging strategies. Research with specific populations of bottlenose dolphins and killer whales (*Orcinus orca*) found that these populations have different hunting strategies to accommodate foraging challenges faced within natural habitats. For example, a population of dolphins in Shark Bay, Australia, developed a sponging foraging strategy in which some of these dolphins remove sponges from bottom attachment to place on their rostrums to forage in deep areas with buried or partially buried fish [2,40]. Animals from this population later developed another foraging innovation involving shells in which they trapped fish [41]. Additional investigation within this dolphin population suggested that these foraging strategies were both genetically and observationally transmitted among the population [42,43,44,45]. Some adult females and their daughters were more likely to utilize these strategies [42,43,45], which may suggest a possible sex difference in innovation [40,44,46].

Another example of sex-based foraging strategies was observed with different killer whale populations that beach themselves to catch penguins, seals, or sea lions [47,48,49]). In one population, only females beach themselves [47,48], whereas in another population males and females beach themselves [49]. While two populations of bottlenose dolphins also display a beaching technique to chase fish onto mud flats, sex differences have not been reported (i.e., strand feeding [50,51,52]). Dolphins in different geographic regions utilize other foraging strategies including mud plume, mud ring, rounding up bait balls, fish stunning, among many others [1,45,53,54], although no sex differences have been identified. Although these strategies are solving a functional problem, the variability in solutions suggests innovation and cognitive flexibility are present within delphinids, if not odontocetes generally.

#### 1.3.2. Trained Creativity

Creativity in animals has also been conditioned through reinforcement procedures (for a recent review, see [39]). Early efforts in training an “innovate” cue with rough-toothed dolphins (*Steno bredanensis*) was met with individually variable success, but the training ultimately produced “original”, never trained, or never before observed behaviors that were eventually put under stimulus control using distinct discriminative stimuli [38]. Asking animals to “innovate” or “create” while under stimulus control has since been expanded to many other non-human species and has been used as a behavior to cognitively stimulate a variety of animals (e.g., killer whales, dolphins, dogs, human preschoolers [39,55]) to assess memory for recent actions [56,57], to add new behaviors to a trained repertoire [34], to provide experience to facilitate problem solving [58], and to assess the degree of creativity abilities (for a review [59,60]; for specific applications [5,37]). These studies and others [61,62] demonstrate the short-term memory (STM) capabilities of these animals, with only two studies systematically investigating the parameters of dolphin STM within the “innovate” context [56,57].

### 1.4. Measures

When creativity is measured for humans, most researchers distinguish between convergent and divergent creativity [63]. Convergent creativity is typically defined as producing one solution that is unique but functional, whereas divergent creativity is composed of creating many different solutions that may or may not be functional. The shelling behavior in the Shark Bay dolphin population is a unique foraging solution that is also functional and may represent a convergent solution [41]. In contrast, a population of dolphins that exhibits myriad foraging solutions may be representations of divergent creativity [41,45,64]. In 2004, Kaufman and Kaufman [60] proposed that behaviors produced by animals under stimulus control could be measured for four constructs typically used to describe human creativity: fluency (number of responses given), flexibility (variation in the types of responses), elaboration (degree of details in a response), and originality (degree of novelty of response). These four constructs, originally conceived by Guilford in 1966 [65] and later quantified in the Torrance Tests of Creative Thinking (TTCT [66]), have provided the foundation for studies investigating different aspects of creativity, including the influence of extrinsic rewards on creative behavior [67,68]. As reviewed by Eisenberger et al. [67], studies have shown that while extrinsic reinforcement will increase creative output (fluency) and the degree of novelty in output exhibited (originality), removal of the external reward will decrease performance eventually, although with individual variability. Studies on creativity in controlled environments (i.e., standardized tests like the TTCT) or with extrinsic motivation suggest that individuals vary in their overall degree of creativity, as measured using the four established constructs. While human research suggests that creative tasks can be cognitively engaging and elicit positive emotions [69,70], *systematic* studies in which animal creativity and perceived cognitive engagement have been experimentally studied are absent. Anecdotally, reports on animal engagement during sessions in which the “innovate” or “create” behavior is asked suggests that the animals are engaged, excited, and willing to persist despite errors [5,37,71].

### 1.5. Purpose

The purpose of the current study was to examine individual dolphin responses to a trained discriminative stimulus to expand previous research on this topic with other small delphinids [5,38,56,57]. A secondary purpose of the study was to explore the role of learning a trained concept as an example of cognitive stimulation. Conducted with a population of bottlenose dolphins housed in a naturalistic social structure and lagoon setting, 12 dolphins (juvenile to adult age ranges) were trained on a “create” discriminative stimulus in which the dolphins learned to emit different behaviors to each request. As described by Kaufman and Kaufman [60], the degree of creativity in bottlenose dolphins was measured in the current study by applying constructs operationalized from a human-based creativity assessment developed by Torrance [66], the Torrance Tests of Creativity Thinking (TTCT). Hill et al. [37] evaluated the four constructs of creativity (fluency, flexibility, elaboration, and originality) for killer whales and found that the different constructs could be assessed reliably using definitions modified for non-verbal, non-human animals, despite the concerns Kaufman and Kaufman [60] described regarding elaboration. Given that previous studies with bottlenose dolphins assessed different aspects of three of the constructs, the current study represents the first opportunity to evaluate all aspects of creativity with the largest bottlenose dolphin sample to date. Using the outcomes of these constructs, we also evaluated individual profiles of creativity for each dolphin. Finally, we consider the use of this trained concept as a form of cognitive enrichment within the context of the individual constructs. The following questions were assessed:

1. How much variation in creativity do bottlenose dolphins show while under stimulus control?
2. Do dolphins show individual creativity profiles?
3. Is there evidence that learning this task is cognitively stimulating?

## 2. Materials and Methods

### 2.1. Subjects

Twelve bottlenose dolphins were included in this study from Roatán Institute for Marine Sciences (RIMS), Roatán, Honduras (Table 1). Seven males and five females were tested, ranging in age from 7 years to estimated 30 years (Table 1). The facility is a natural habitat composed of a large lagoon (~8000 m^2^ surface area) with six adjacent habitats of varying sizes and depths (ranged from beach to ~7.5 m, with a tidal fluctuation of ~0.5 m) separated by gates. The six habitats are used for temporary social separations for facility operations and were used for training and testing for this research study. The social composition fluctuated between 17 and 22 dolphins of mixed age and both sexes who live together in this large lagoon habitat, simulating a typical bottlenose dolphin social structure. Male dolphins were trained and tested in two of the smaller adjacent pools with those results described in greater detail in Melzer et al. [55]. Female dolphins were trained and tested in the main lagoon or in an adjacent habitat using the same procedure as the males. Experience with the “innovate” concept varied across each dolphin due to daily facility activities. This behavior was originally introduced as a form of cognitive enrichment and was considered for research purposes at a later time. Thus, experience with the concept was not documented systematically or trained systematically. See Dudzinski et al. [39] for additional details regarding the habitat at RIMS and the history of this concept.

### 2.2. Measures

Following the definitions established in Melzer et al. [55] and expanded upon by Hill et al. [37], creativity was measured for four variables: fluency, flexibility, originality, and elaboration (summarized in Table 2). These variables were derived from the Torrance Tests of Creative Thinking (TTCT, [66]), as originally proposed by Kaufman and Kaufman [60] and expanded upon by Kaufman [59]. Each variable was operationalized using two to four definitions (see Table 2 in Hill et al. [37]). A brief description for each operational definition follows for each variable. The criteria for scoring behaviors as correct or incorrect is presented in Section 2.3.1.

Fluency was defined as the number of correct different behaviors presented within a session with four different operational definitions assessed: (1) total number of reinforced (correct) behaviors, (2) number of reinforced (correct) behaviors from the total number of behaviors performed (percent correct), (3) total reinforced and non-reinforced behaviors performed, and (4) highest number of consecutively reinforced behaviors before a behavior was repeated and not reinforced [37]. For this study, we evaluated the percent correct (2) and number of trials before repeating (4) as two separate measures of fluency.

Flexibility was defined using operational definitions for two different measures: (1) energy and (2) type. Energy was divided into 10 different levels, beginning with low (L, level 1, e.g., most vocals or brief pectoral fin movement), moderate (M, level 2, e.g., spy hop, low energy swims, double pectoral fin movement), and high (H, level 3, e.g., aerials of any kind, high speed swims), followed by different combinations of compound actions (levels 4–10); energy was determined by the degree of effort or intensity needed to emit the behavior (4—2 L homogenous actions, 5—2 M homogenous actions, 6—2 H homogenous actions, 7—L + M heterogenous actions, 8—L + H heterogenous actions, 9—M + H actions, 10—L + M + H heterogenous actions; see full description in Hill et al. [37]). Flexibility type was differentiated into single locomotor (L, level 1), single vocal (V, level 2), or single bubbles (B, level 3) behaviors, with different combinations of those categories divided into the remaining levels (levels 4–10) for a total of 10 different levels (4—2 L double actions, 5—2 V double actions, 6—2 B double actions, 7—L + V double actions, 8—L + B double actions, 9—V + B double actions, 10—L + V + B multiple actions; see full description in Hill et al. [37]).

Originality was represented by four behavioral measures: (1) behaviors that only one dolphin exhibited compared to other dolphins (i.e., unique), (2) a behavior that was emitted a single time across a dolphin’s test sessions, but other dolphins may have also exhibited the behavior (i.e., single), (3) a behavior that was produced only by one dolphin and performed only once across all sessions (i.e., true original), and (4) a behavior that was invented during the test sessions, not previously in the dolphin’s stimulus-controlled repertoire (i.e., such as different types of bubbles released, invented).

Following the operational definitions established for research on responses to the innovate cue by killer whales [37], elaboration was measured based on the presence of a single behavior (e.g., aerial, vocal, swim, body part movement, or bubbles) or multiple behaviors that were performed consecutively (e.g., sink followed by a bubble) or simultaneously (e.g., sink while exhaling and blowing bubbles).

### 2.3. Procedure

#### 2.3.1. Training Procedure

The concept of innovate was trained intermittently across all subjects, beginning in 2005 as a cognitive enrichment behavior. Training sessions occurred on average 1–2 times a week across multiple months for each dolphin individually, with longer breaks to work on other training tasks or calf care. Across all 12 dolphins, total exposure to training this concept ranged from less than a year to over seven years depending on the individual. Two males, Lenca and Champ, and two females, Poli and Tilly, had less than five years of training on this task. The remaining five males and three females had been exposed to the task for more than five years (Table 1). Initial training and training for test sessions was performed by a primary trainer (TB) for all dolphins; and all dolphins had an additional trainer who maintained the behavior intermittently across the years. Dolphins were rewarded differentially on every trial, if they exhibited highly different behaviors from trial to trial (vocal vs. jump vs. underwater behaviors), using magnitude of primary reinforcement (fish: capelin and/or herring) and secondary reinforcers (clapping, dancing, cheering). If dolphins performed a similar type of behavior that was different than the immediate previous trial and previous trials, a smaller magnitude or reinforcement (1 capelin) or a lower energy secondary reinforcement (e.g., whistle bridge or clap) was provided. Dolphins were hinted with different trained behaviors throughout the training process. Additional details regarding the general training method used to train the innovate behavior can be found in Dudzinski et al. [39].

For this study, acceptable training criteria for a correct response included a single action that was different from the previous behavior emitted and had to be performed immediately after the S^D^ was given (within 10 s). That is, dolphins could not add on an additional action to a behavior performed previously or while waiting for the bridge (e.g., sampling of behaviors was not acceptable for correct responses) for each cue. Training efforts were coordinated across a two-year period to enable formal testing of the knowledge of the “innovate” concept during the researchers’ field seasons. Frequency of training sessions was increased per week for a 3–4 month period prior to each field season in which the principal investigators were able to be present for test sessions. Acceptable criteria for moving to testing were each animal had to respond correctly (not repeat a behavior already performed in the session) for 4–5 trials in a session that was intermixed with other behaviors. Most “innovate” training sessions were short, and some additional training had to be conducted to extend the sessions as one of the measures for fluency was the number of trials an animal could perform successfully within a session. Finally, each animal had their own timeline for learning the “innovate” concept and thus had different seasons of data collection.

#### 2.3.2. Test Trials

Data were collected at three separate times: 8–12 January 2018, 14–19 January 2019, and 3–7 May 2021, which corresponded with field seasons and dolphin training of this concept and ranged between 5 and 43 trials across sessions. As detailed in Melzer et al. [55], two video camera set ups were used to capture multiple perspectives: one person on the dock next to the trainer with a Go-Pro Hero 4 and a stationary swimmer with two Go-Pro Hero 3 cameras positioned at the surface and underwater. The cameras recorded the entire session and enabled recording of all behaviors above and below water. Up to seven test sessions were conducted for individual dolphins, but only sessions 2–5 were analyzed for the project. The first session for all animals was used as a warm-up to transition to the testing phase. Test sessions differed from training sessions in several ways: (1) the number of people around the habitat increased from one trainer to five humans (trainer, underwater camera operator, dockside camera operator, session transcriber, and research observer), (2) the criterion for ending a session occurred when the dolphin performed three incorrect responses consecutively, (3) no hints such as using hand gestures for known behaviors could be provided, and (4) a variable number of trials was conducted. Lenca participated in only four sessions due to timing of research sessions and interest in this activity. To be consistent with the other dolphins, the first session was not included and a “fifth” session was imputed using the mean of the remaining three sessions available for all variables of interest to facilitate analyses. One test session per day was used for this study per dolphin.

As described in Melzer et al. [55], an “innovate” test session began with dolphins given the innovate S^D^, a hand gesture, by the primary (and only) test session trainer (T.B.). No “warm up” behaviors were given. A trial began with the execution of the innovate S^D^. This procedure was the same for the training sessions conducted during the two-year period in preparation for testing. The trainer determined if the behavior was different from other behaviors presented in previous trials within the session. If the behavior was different (i.e., not the same behavior performed previously regardless of energy, the same behavior with a different behavior performed simultaneously or within 1–2 s of a previous behavior, or a new/novel behavior never performed by the dolphin), the dolphin was correct and bridged with a whistle and given a primary reinforcement of fish. Magnitude of reinforcement was held constant (2–3 capelin or 1 herring) to avoid cueing and training of the concept during test sessions. Dolphins had 4–6 lbs of fish available for test sessions, depending on age and size of dolphin. If the dolphin repeated a previous behavior performed within that session, the trainer sat neutrally in front of the animal (i.e., a least reinforcing stimulus or LRS [72]) and then gave a new innovate S^D^. Test sessions ended when a dolphin presented the same behavior three times in a row, following the first time the behavior was emitted in a test session. To address possible frustration after three failures, the “innovate” test session was ended and the trainer moved on to other behaviors that could be reinforced. A session transcriber recorded all emitted responses and administered consequences for each trial of a test session.

#### 2.3.3. Coding

A two-step process was conducted to code the data: video coding and data scoring. All dolphin test sessions were videotaped and later coded by independent research assistants to verify that the behavior that was emitted by the dolphin and reinforced using a whistle bridge and fish, or not, by the trainer matched the documented behavior by the session transcriber (H.M.H.). All trials were assessed by two independent researchers: original session transcriber (H.M.H.) and video-recorded session coder (D.B.Y.). Dolphin responses that were repeated later in a session that had previously been reinforced with primary rewards were noted in the scoring process, but were not considered correct for the test sessions. That is, behaviors that received secondary reinforcements, such as claps or praise, were not coded as correct for this study but were counted towards the total number of trials given in a session. Agreement was 100% between the video coder and the original session documentation.

Three research assistants (RAs) scored each correct emitted behavior per trial within each test session for each of the operational definitions defined in Section 2.2 for the four creativity variables assessed within the Torrance Tests of Creative Thinking (TTCT, [66]). That is, for each trial, research assistants scored the four fluency operational definitions, the two flexibility operational definitions, the three elaboration definitions, and whether or not the behavior was truly novel. The other three originality definitions were scored by frequency of response within a dolphin, across all sessions, and across all dolphins and sessions. The male dolphin correct trials were scored by one RA (G.G.), and the female dolphin trials were scored by one RA (M.R.). A third RA (K.S.) assessed 25% of the available trials for both males and females for reliability and was in agreement with the original coders for 95% of the variables of interest using an inter-rater reliability of agreements/(agreements + disagreements).

### 2.4. Statistical Analyses

While the current sample size was larger than previous studies [5,56], it is still considered limited with seven male dolphins and five female dolphins and varying years of training experience on this concept. As with the killer whale creativity study [37], frequency data were standardized for some operational definition measures (i.e., fluency percentage correct and flexibility measures) to facilitate comparisons across and between dolphins by converting to proportions (e.g., frequency counts per flexibility level were divided by total number of reinforced trials). When possible, we also provided individual results for the 12 dolphins. Descriptive statistics are presented for all results. Individual dolphin scores were rank ordered using z-score calculations for each creativity construct to create a creativity profile for each dolphin. Before this ranking could be performed, we had to validate the measures that would be used for the final rankings. The validation process is described in the supplemental material. Exploratory correlational analyses were conducted to begin to assess cognitive stimulation of the “innovate” behavior between sessions and construct operational definitions.

#### 2.4.1. Validation Measures

Multiple measures were calculated to validate the profile created for each dolphin within each construct. For example, originality was scored on three different operational definitions, these definitions were then aggregated in different ways to create an originality profile across dolphins. The procedure utilized to calculate the validation measures may be found in the Supplemental Material. Final scores for each validation measure are summarized within the supplemental tables.

#### 2.4.2. Individual Construct Overall Rankings

Depending on the construct, summed z-scores that were calculated for most of the validation measures for each construct were utilized for this ranking process. All dolphins were rank ordered for each construct.

#### 2.4.3. Overall Creativity Score Rankings

Using the summed z-scores from the individual constructs, we then created an average for each construct that summed the z-scores across the total number of operational definitions assessed for each construct and then divided that number by the number of operational definitions used within the construct. Fluency was calculated by summing the four z-scores and dividing by four. Flexibility was calculated by summing the two z-scores and divided by two. Originality was calculated by summing the three z-scores and divided by three. These three averages were then summed and rank ordered.

## 3. Results

The results of the 12 dolphins are divided into each construct with overall details and results from inferential statistical tests for each operational definition of interest followed by a presentation of a creativity profile for each animal and across animals. This section ends with results from exploratory analyses conducted to assess cognitive stimulation.

### 3.1. Variation across Constructs and Individual Differences

Each dolphin responded distinctly during the creativity test sessions. Individual dolphins produced a different number of trials per test session because of their individual responses to the innovate cue (i.e., some animals made three errors in a row sooner than other animals). Table 3 and Table 4 summarize the responses of each dolphin per session for all variables of interest divided by sex to facilitate data presentation. Across the four sessions conducted for each dolphin, 5–43 trials were completed with an average of 21 trials completed in a session (Table 3 and Table 4). The supplemental file provides the graphical representation for each individual dolphin per construct of interest.

### 3.2. Fluency—Percent Correct

**Figure 1:**
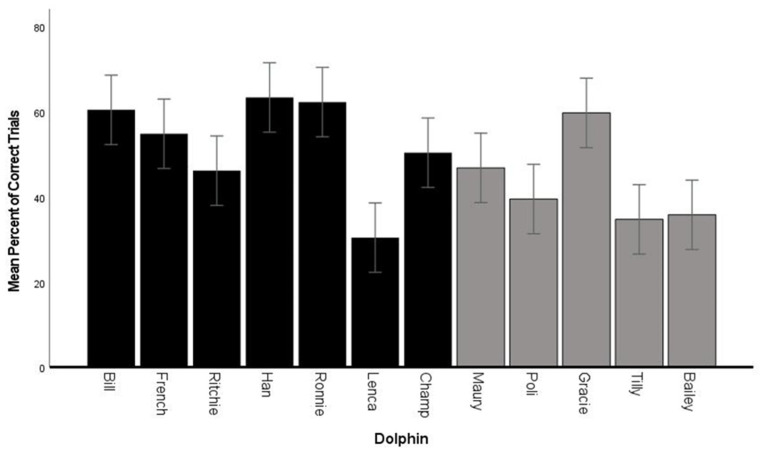
Mean overall fluency performance for each dolphin. Error bars are ± 1 SE. Black bars are male. Gray bars are female.

Overall, the 12 dolphins produced a fluency (percent correct behaviors) average of 47% (SD ± 18%) with a range between 8 and 100% (Figure 1, Table 3 and Table 4). Han was correct for 63% of his trials (67/107), followed by Ronnie with an overall performance of 59% (62/106), Bill at 57% correct (59/103), and French at 53% (48/90) (Figure S1). All other animals (*n* = 8) had reduced fluency in their overall performance (Figure 1 and Figure S1), possibly demonstrating partial mastery of the task based on this measure.

### 3.3. Fluency—Number of Trials before Repeated Behaviors

**Figure 2:**
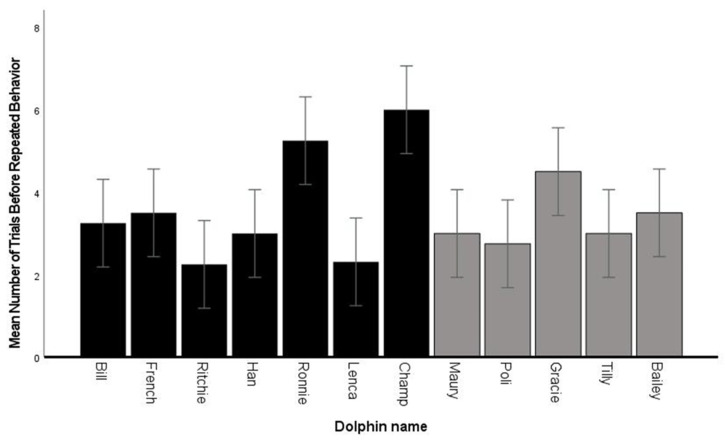
Mean number of trials before repeated behavior by each dolphin. Error bars are ± 1 SE Black bars are male. Gray bars are female.

Overall, the 12 dolphins produced a fluency average of 3.4 (SD ± 1.1) before a behavior was repeated incorrectly, with a range between 1 and 12 (Figure 2, Table 3 and Table 4). Champ performed the most consistently across test sessions with an average of six different behaviors before repeating the same behavior immediately after the first time it was performed (Figure S2). Ronnie produced the greatest number of behaviors (*n* = 12) before repeating a behavior in Session 2; this session was an outlier compared to his other sessions (Figure S2).

### 3.4. Flexibility—Energy

**Figure 3:**
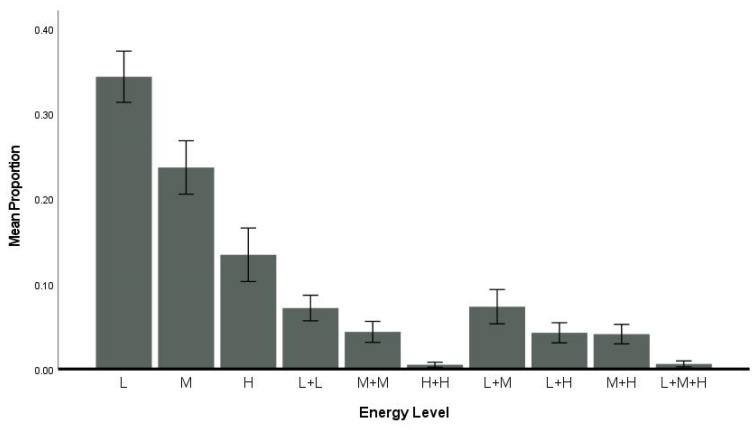
Mean proportion per energy level across all dolphins. L = low energy, M = moderate energy, H = high energy, L + L = homogenous L behaviors, M + M = homogenous M behaviors, H + H = homogenous H behaviors, L + M = heterogenous L + M behaviors, L + H = heterogenous L + H behaviors, M + H = heterogenous M + H behaviors, L + M + H = heterogenous L + M + H behaviors. Error bars are ± 1 SE.

On average, dolphins exhibited responses with different energy levels (Figure 3; Table 3 and Table 4), with single behaviors with low energy comprising the highest proportion of responses across trials (*M* ± SEM, 0.34 ± 0.03), followed by moderate energy single behaviors (0.24 ± 0.03), and high-energy single behaviors (0.13 ± 0.03). Same or mixed energy level behaviors (homogeneous low: 0.07 ± 0.02, mixed low and moderate: 0.07 ± 0.02, and all other combinations, Figure 3) occurred less often. As summarized in Table 3 and Table 4, individual dolphins varied in their energy levels and the use of a combination of behaviors with multiple energy levels, with eight dolphins showing greater diversity in their exhibited behaviors across correct trials: Bill, Ritchie, Han, Ronnie, Champ, Maury, Poli, and Bailey (Figure S3).

### 3.5. Flexibility—Type

**Figure 4:**
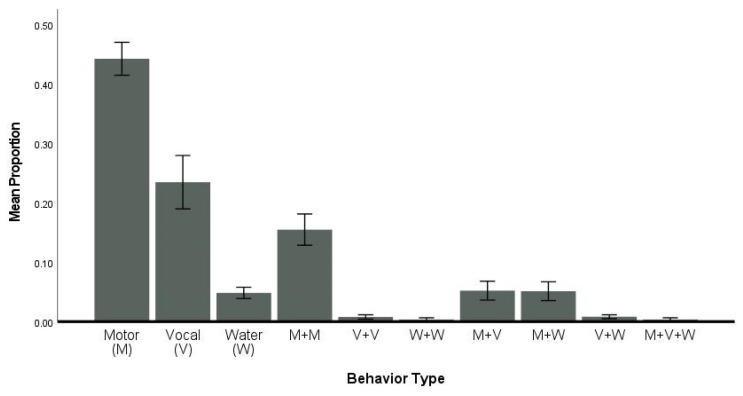
Mean proportion per type of action across all dolphins. Error bars are ± 1 SE.

Dolphins responded primarily with four types of flexibility: motor (type 1), vocal (type 2), multiple actions both/all motor (type 4), and water actions (type 3) (Figure 4; Table 3 and Table 4). Dolphins exhibited single motor behaviors with the greatest proportions compared to other single action (vocal or water), any combination of two or more behaviors, or a combination of all three types of behaviors. Dolphins also produced more single vocal behaviors than any other combination of multiple actions, except for motor–motor combinations. Dolphins rarely displayed water actions or any combination of multiple actions, but single water actions were performed more frequently than double water, double vocal, and water and vocal (Figure 4). As summarized in Table 3 and Table 4, individual dolphins varied in their type of behaviors and the use of a combination of behaviors from different types, with six dolphins showing greater diversity in their exhibited behaviors across correct trials: Maury, Han, Champ, Tilly, Bill, and Bailey (Figure S4).

### 3.6. Originality

**Figure 5:**
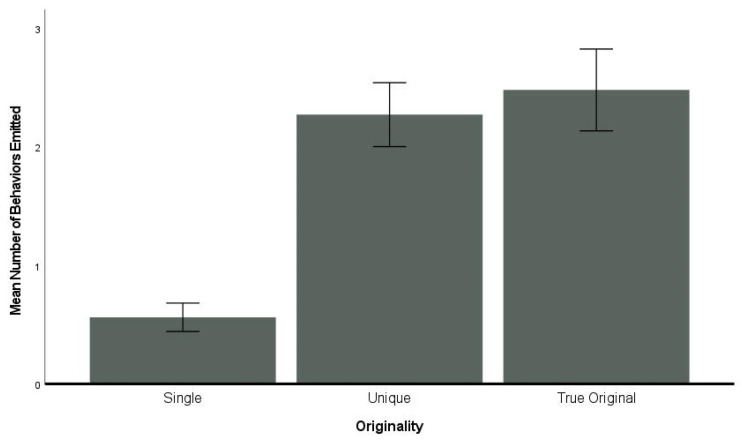
Mean number of behaviors emitted per originality category. Error bars are ±1 SE.

All dolphins produced primarily single actions but did exhibit complex actions (Table 3 and Table 4). On average, the frequency of unique behaviors (*M* ± SE, 0.56 ± 0.12) was lower than either single (2.27 ± 0.29) or true original behaviors (2.48 ± 0.39). Individual variability was observed across the males and females (Figure 5 and Figure S5).

Four animals produced four different behaviors that were not under stimulus control at the time of testing that could be considered truly novel: Bill released three bubbles in slow succession; French released one slow bubble; Ronnie released several small bubbles in quick succession; Tilly exhibited a lay back with her tongue hanging out, which included a combination behavior of a known behavior (layback) and a spontaneous behavior (tongue hanging out). In general, however, the majority of the behaviors selected by the dolphins during their responses were behaviors within their trained repertoire.

### 3.7. Elaboration

**Figure 6:**
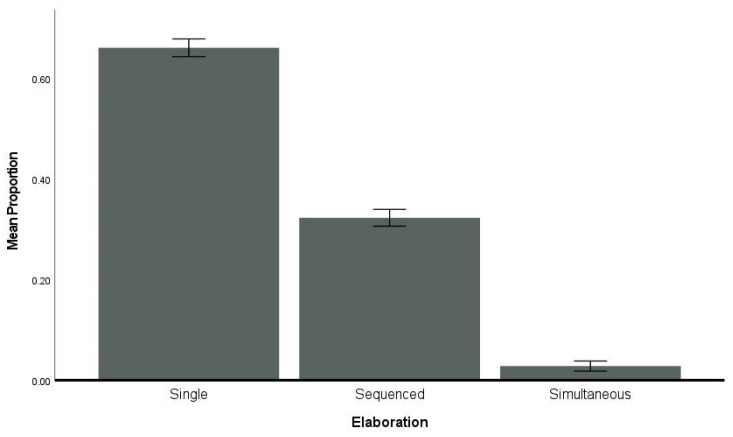
Mean proportion per elaboration category. Error bars are ±1 SE.

All dolphins presented larger proportions of single behaviors (*M* ± SD, 0.66 ± 0.02) than multiple actions (sequences, 0.32 ± 0.02, or simultaneous, 0.03 ± 0.01) in response to the innovate cue. Individual variability was observed (Table 3 and Table 4; Figure 6 and Figure S6).

### 3.8. Profile of Creativity Based on Z-Scores

Three measures for fluency were calculated for validation purposes—summed ranking, summed z-score, combined total score (see Supplementary Materials). All three measures generally aligned with the dolphins who scored the highest on fluency with a few variations. The top four dolphins that generally completed more trials with more correct responses were Champ, Ronnie, Han, and Bill. Dolphins that consistently completed fewer trials with fewer correct responses were Tilly, Lenca, Bailey, and Poli (Table 3, Table 4 and Table 5).

Three measures of flexibility were calculated for validation—unweighted sum, weighted sum, summed z-score. All three measures generally aligned for the dolphins who scored the highest on flexibility with a few variations. Han was consistently ranked as the top for all three validation measures of flexibility. French ranked second on both weighted scores. Champ ranked third on the unweighted and summed z-score. Bill was second for unweighted sums and third for weighted sums. At the bottom, were Gracie and Lenca for flexibility across all three validation measures (Table 5).

Three measures of originality were calculated for validation—total score, weighted score, and summed z-score. All three measures generally aligned with the dolphins who scored the highest on the originality construct with a few variations. The dolphins that exhibited “original” behaviors most consistently were Han, Bill, and Ronnie, with Gracie, Lenca, and Bailey exhibiting the least “original” behavior rankings (Table 5).

A holistic score of creativity was calculated from the summed averaged z-scores of the three constructs (i.e., fluency, flexibility, originality). The results of this overall score showed that the animals ranked at the highest rankings also consistently ranked high in the individual measures (Table 6). The animals with longer, more consistent training histories tended to be top ranked individuals, except for Champ, whose training history (~3–4 y) was not as long as the adult males (i.e., Han, Ronnie, Bill with >5 y). This pattern was supported by the females who tended to be ranked lower in overall creativity. Maury was an exception, as she ranked higher than all of the females and some of the males (Ritchie and Lenca); however, Maury had more training than many of the females (~5 y). Individual differences were observed for specific animals in each construct that are worth noting. For example, Champ, who had less training than many of the other dolphins, was high for fluency and flexibility but in the bottom half for originality. Maury, who had more training experience, was in the top four for flexibility but middle of the rankings for fluency and originality. Lenca, who had the least experience for males, besides Champ, and less experience than the three oldest females was at the bottom of all categories.

### 3.9. Cognitive Stimulation

Exploratory correlational analyses were conducted for accuracy within fluency. We expected the number of trials over sessions would increase if the “innovate” concept was cognitively stimulating; that is, dolphins would produce more trials over the course of several sessions as a proxy for cognitive stimulation. No significant or clear relationship emerged from this analysis at either the group or individual dolphin levels. Rather, fluency accuracy remained relatively stable across the sessions for individual dolphins. However, the dolphins did continue to persist with the session despite errors occurring. We also assessed whether truly unique (novel) behaviors occurred more frequently over sessions. Again, no relationships were found for the frequency of truly unique behaviors at the group or individual levels. However, more different types of behaviors were produced in the last session than in the previous sessions on average, which may represent cognitive facilitation or enrichment. Finally, although the dolphins did not have many opportunities to combine behaviors into simultaneous or sequenced actions, these more complex interactions were observed as test sessions continued.

## 4. Discussion

Teaching animals to be “creative” has been successful across many species using a variety of different training methods (reviewed by Dudzinski et al. [39]). Karen Pryor originated this idea with rough-toothed dolphins in the early 1960s [38]. Many trainers have trained their animals on this concept as a form of enrichment or as an opportunity to expand behavioral repertoires [39,58]. However, some researchers have applied human-based creativity constructs (fluency, flexibility, elaboration, originality) to evaluate creativity itself in the behavioral outputs of animals under stimulus control, following a suggestion by Kaufman and Kaufman [60]. Studies with marine mammals in managed care have examined aspects of fluency, flexibility, and originality [5,37,55]. Elaboration, as Kaufman and Kaufman [60] suggested, may be a little more difficult to measure reliably. Research with killer whales found that elaboration could be measured, but it appeared to be dependent upon opportunities to add on behaviors, such as a chained response [37].

The current study expanded previous work with bottlenose dolphins by increasing the sample size and adding more females to the pool of animals assessed on the four creativity constructs [5,38,56]. This study also utilized stringent test criteria such that the dolphins were unable to chain multiple responses together if they began a chain with a behavior that had been produced previously within the session (allowable criteria in other studies as reviewed by Dudzinski et al. [39]). As expected from previous studies with dolphins [5,37,55], the 12 dolphins (7 males, 5 females) in this study exhibited variation in their “creativity” while under stimulus control as evidenced by individual differences that emerged across each of the four constructs as well the “creativity profile” that was created for each dolphin as a novel contribution to the literature. As the individual profiles suggested, the creativity constructs were most likely influenced by training experience, innate creativity, individual differences, life experiences, or some interaction between these variables. Ultimately, as described by trainers who have worked on this concept with different species, this behavior is engaging and stimulating for the animals [5,37,55,56,57,58].

### 4.1. Fluency

The 12 dolphins averaged 21 trials per session, with an overall performance of 47% correct. Animals ranged between 8% and 100% correct in individual sessions, suggesting that dolphins were variable in their performance within a session. On average, the dolphins produced 3.4 different behaviors before they repeated one of their previously emitted behaviors (“same”). One adult male produced 12 different behaviors in consecutive trials before he repeated for the first time. However, the youngest male dolphin with the least experience averaged six different behaviors before repeating a same behavior. Together, these results indicate that the majority of the dolphins mastered the concept and were reliable in producing different behaviors in a session. The two animals that performed the worst may have needed more experience with the task or perhaps were not as engaged in the task as others. When compared to the previous studies with dolphins in which accuracy was evaluated (three adult male bottlenose dolphins [5] and nine male and female killer whales [37], the performance of the current subjects was below the fluency performance. This difference in performance (above 85% for both genera of delphinid vs. about 50% for the current sample) was most likely due to training differences and different criteria for “correct” behaviors; specifically, the dolphins in the current study were not allowed to “add on” behaviors to a behavior they had previously performed (i.e., the trainer bridged the animal immediately after a behavior was exhibited). There were occasions on which a dolphin performed a sequence of behaviors before the trainer could bridge and these actions were correct if the behaviors performed were different from previous behaviors.

### 4.2. Flexibility

Flexibility was measured with two operational definitions that were similar to those used in the previous studies with bottlenose dolphins, killer whales, human preschoolers, and dogs [5,37,55,58]. The first operational definition evaluated whether dolphins produced behaviors with different energy levels. The previous studies found that all species tended to prefer low energy with some moderate-energy single-action behaviors; however, there were individual differences with some individuals preferring high-energy behaviors or showing a high degree of variation in energy for the behaviors emitted [5,37,55,58]. The bottlenose dolphins in the current study followed this trend, with most actions produced categorized as low-energy behaviors. Thus, the dolphins would produce a pectoral fin raise, a roll, a head nod, a vocal, or bubbles, which are all low-energy behaviors rather than a jump, fast swim, or a combination of multiple behaviors with different energy levels. The second definition considered the type of behaviors the dolphins selected in their response to the S^D^, which were categorized as motor, vocal, or water and different combinations of these. Like the previous studies [5,37,55,58], the dolphins in the current study also displayed single-action motor behaviors followed by single-action vocal behaviors, and combinations of two motor actions over and above any water manipulation behaviors. Despite the more stringent behavior criterion for single-action behaviors in the current study, the dolphins did produce combination behaviors, which suggests that they were capable of more flexible thinking. These combination behaviors included both sequential behaviors in which two behaviors were performed in sequence before a bridge was given or simultaneously before a bridge was given. Individual variation was also observed across the flexibility construct with mainly adult males, except for Champ, a juvenile male, with higher flexibility scores as compared to the adult females and the other juvenile male, Lenca, who had the least degree of flexibility across the 12 dolphins. It is possible that this variation in flexibility reflects individual differences in overall creativity, different levels of understanding of the task, or different amounts of training experience. This construct may underly the innovation in foraging styles exhibited by dolphins that perhaps should be considered as a “variation on a theme”. For example, a few dolphins in Shark Bay first exhibited a foraging style in which they placed sponges on their rostrums while finding prey [2]. This behavior was transmitted socially within one matriline and across other members of the social group [40]. Several years later, a subset of this population replaced sponges with shells as they foraged, presumably for a similar function [41].

### 4.3. Originality

The sponging foraging behavior was invented by a specific female dolphin in Shark Bay [40], and to our knowledge, has not occurred in other populations. Atlantic bottlenose dolphins off the coast of South Carolina have been observed beaching themselves onto mudflats in pursuit of fish that have been driven there by dolphins [50]. Similarly, two different, geographically isolated killer whale populations have invented slightly different beaching techniques in which individuals pursue penguins, seals, or sea lions onto beaches [47,48,49]. These foraging examples illustrate that delphinids are not only curious, but inventive when faced with a foraging challenge. Originality, or the expression of truly novel or unique behaviors, is typically considered a hallmark characteristic of creativity [65]. Pryor originally demonstrated that rough-toothed dolphins could display truly novel behaviors while under stimulus control when she first introduced the concept of “innovate” as a trained behavior [34,38]. We assessed several operational definitions of originality that were informed from earlier work [5,34,37,55] while keeping the human-based criteria of originality in mind [59,60]. Three of the four definitions of originality examined in the current study focused on absolute frequency of the behaviors emitted by the dolphins, depending on the definition and the frame of reference (individual, sessions, or all dolphins and sessions). The dolphins emitted almost 2.5 different behaviors, on average, that occurred only once in one dolphin across all trials, sessions, and dolphins (i.e., true original) and just over two different behaviors, on average, in which the behavior was performed by an individual across all sessions and trials one time but may have been performed by other dolphins too (i.e., single). Finally, behaviors that were exhibited by only one dolphin across all trials, sessions, and dolphins, but that dolphin may have produced it multiple times (i.e., unique) were less likely to occur (average rate of 0.55). Together, the results from these three operational definitions suggest that dolphins produced behaviors that were unique to them and did not necessarily produce behaviors that other animals also exhibited. Given that the dolphins were tested independently and in separate locations, social contagion should not have influenced these results. For dolphins in managed care who have been trained for many years, the most difficult aspect of this construct is “knowing” whether an individual has produced a behavior before and if it is thus truly novel. To address this issue, the training history and behavioral repertoire must be known, which was not fully possible with the current study. Ultimately, only four *novel* behaviors, or original behaviors not under stimulus control, were displayed during testing by four different animals, as the remainder of the behaviors displayed were part of the dolphins’ trained behavioral repertoire. Curiously, Tilly, the worst-performing dolphin in most of the constructs, showed the greatest novelty in her behavior, as she combined a known behavior with a spontaneous behavior in one of her sessions. This layback with tongue hanging out was truly original across all the definitions. Most likely, these results are influenced by the protocol utilized during training to minimize chained behaviors, and while Tilly had some experience, she clearly still had more to learn regarding the concept of innovate, although one might argue her novel display of creativity was due to her limited training on the specific task parameters.

### 4.4. Elaboration

Kaufman and Kaufman [60] suggested that elaboration would be very difficult to evaluate for non-human animals. We believe that it can be operationalized and reliably coded but *is* highly dependent on the training protocol and the testing criteria that are accepted. Specifically, if animals can chain together behaviors as they are learning the concept, elaboration is more evident than if animals must perform completely different behaviors in each trial, as was the goal in the current study. The initial studies with both bottlenose dolphins and killer whales allowed the animals to “add on”, which elicited more combination behaviors with either sequenced actions or simultaneous actions [5,37]. Given the stringent criteria established for the current study, the dolphins did not have a strong reinforcement history of combination behaviors and as a result, elaboration was not included in the creativity profile scores. Therefore, the most frequently occurring category for elaboration was single behaviors. The dolphins did engage in combination behaviors with sequenced actions more frequently than simultaneous actions. Sequenced actions occurred in about 30% of the correct trials. Simultaneous actions occurred rarely, although some animals did produce them, as the example of Tilly above demonstrated. Anecdotally, when some of the originally tested dolphins received additional training on this concept with different accepted criteria, the dolphins started producing more simultaneous and sequenced behaviors, suggesting that training history strongly influences the degree of elaboration exhibited.

### 4.5. Overall Creativity

After examining each of the constructs, it was clear that while some animals were consistently high or low on the constructs when ranked, other animals differed in their rankings, depending on the construct itself. Overall, dolphins with the most training history on the concept of “create” had the highest rankings across the three constructs aggregated for this assessment: fluency, flexibility, and originality. Adult males with more than five years of consistent training tended to have the highest rankings (Han, Ronnie, Bill). Champ, the least experienced and juvenile male, however, was uniquely positioned as showing overall high rankings for fluency and flexibility while at the bottom of the rankings for originality. His overall creativity profile suggested that he had learned the concept and could select different types of behaviors, though he was somewhat limited in the diversity of behaviors he could “create” spontaneously. Contrasting to these four males were four females who, with the exception of the other juvenile male (Lenca) with less experience, fell in the bottom half of the creativity rankings in most constructs, most likely related to their intermittent training experience. The only female to perform better than some male dolphins was Maury, who had more consistent training experience than the other females as she had not reared a calf prior to testing for this study and had been participating in this training longer than most of the other females who had reared calves during training. Additional research should be conducted to determine if there is any relationship between overall creativity rankings of these dolphins based on a reinforcement contingency and their spontaneous creativity in foraging, play, or other cognitively challenging contexts, such as problem solving.

### 4.6. Cognitive Stimulation

As summarized in Dudzinski et al. (2018), training the concept of “innovate” or “create” is popular among trainers of many types of animals (cetaceans, pinnipeds, canines) and utilized in several different ways, but probably most often as an opportunity to cognitively stimulate the animal being trained [5,34,37,55,58]. For animals in managed care, cognitive engagement is a critical part of maintaining a positive well-being and addressing welfare needs [12,17]. Animals in managed care have less challenging lives compared to their free-ranging conspecifics and must be provided with opportunities to engage with their environments, conspecifics, and sensory experiences [19,20,21,22,23,24]. Interacting with trainers during training sessions is one opportunity to provide cognitive stimulation as Jaakkola and colleagues [73] found in an opportunistic study with a stranded animal who was being rehabilitated and showed greater activity when engaging with a trainer as opposed to engaging with enrichment alone.

Training the concept of “innovate” can be challenging for both the trainer and the animal, as it requires the animal to spontaneously determine the “rules” of a game using only the feedback provided by trainers through reinforcement contingencies. Too many errors during the early part of training can produce frustration [74], so the trainers must learn to balance mistakes with progress. Pryor and Chase [34] supported this point in their description of training the innovate concept with rough-toothed dolphins as well as suggesting that individual differences must be considered. However, from this study and many others that have been conducted, this conceptual behavior can be acquired and does produce variability across the individuals who learn it. Moreover, despite the errors and presumed frustration, the dolphins in this study (as well as previous studies) sustained their interest in the S^D^ and continued to produce responses even after mistakes. This is especially interesting as the dolphins did not receive variable reinforcement following the different types of behaviors they produced during testing. That is, every behavior performed correctly received the same magnitude of reinforcement. Thus, it is possible that this task became intrinsically motivating for the animals. Additional evidence for this interpretation comes from the fact that while training was intermittent and perhaps more reinforcing due to more variable reinforcement contingencies and session lengths, testing was predictable in the frequency of sessions and reinforcement provided, but not in session length, as more trials were given to try to find the maximum number of trials a dolphin could complete (43 in this study as opposed to over 100 by a killer whale [37]). Moreover, qualitative behavioral observations suggested that the dolphins were interested in participating in these sessions, as shown by behaviors indicating continued motivation, stationing with the trainer, continued emission of behaviors even if not reinforced, the presence of victory squeals, which are associated with reinforcing consequences [36], and the production of some original behaviors within a stringent criterion of correct responses accepted. Although retrospectively determined, quantitative measures suggested that the dolphins’ behaviors remained stable rather than increasing with the number of trials performed or the number of reinforced behaviors. Rather, the evidence for cognitive stimulation may be more about how the dolphins varied their responses (flexibility and originality) rather than the total number of responses emitted, as this variable was most likely controlled by the trainer based on the limited amount of primary reinforcement available, repeats of behaviors, and success.

## 5. Conclusions

The current study investigated dolphin responses to a “create” concept while under stimulus control by a trainer. This study tripled the existing sample of dolphins with the addition of 12 individuals that included both male and female dolphins. Although a limited number of test sessions (*n* = 4) was used for the current study to ensure an equal number of sessions was available for all dolphins and testing reinforcement was consistent, unlike training in which reinforcement varied depending on the behavior emitted, the animals continued to perform behaviors through errors, additional trials, and daily sessions during the testing week. Moreover, this study provided additional empirical evidence of cognitive flexibility and “creativity” in bottlenose dolphins, corroborating previous efforts [5,34,37,55]. A preliminary effort was made to create a creativity profile for each animal, with validation efforts supporting the final rankings of the dolphins both within a construct and across three constructs. While training experience, individual motivation, and personal strategies for success within the training and testing process most likely influenced the degree of variation observed across the 12 dolphins, it does appear that “creativity” varies across individuals at a more fundamental level. Some research on personality in dolphins suggested that human raters endorsed different degrees of creativity for individual animals [75]; however, it is unclear under which contexts this dimension was assessed by raters, and additional research is necessary [76]. Future research should continue to examine the question of creativity as a personality characteristic while also evaluating the impact of extrinsic rewards during both training and testing on overall creativity performance [77]. Additionally, examining creativity under stimulus control should be validated with other contexts of creativity such as problem solving or spontaneous play. Finally, an experimental study investigating the degree of cognitive engagement displayed by individual animals during a create session versus a different type of session should be conducted.

## Figures and Tables

**Table 1:** Age range during testing, sex, and training experience with the innovate S^D^ prior to testing for all 12 study animals.

Dolphin ID	Age Range during Tests (y)	Sex	S^D^ Innovate Experience
Han *	~30	M	>5 y
Bill	17	M	>5 y
Ritchie **	~18	M	>5 y
French	14	M	>5 y
Ronnie	16	M	>5 y
Champ	6	M	<5 y
Lenca	6	M	<5 y
Gracie **	27; 29	F	>5 y
Maury	16; 17	F	>5 y
Bailey	14; 16	F	>5 y
Tilly	8; 11	F	<5 y
Poli	8; 11	F	<5 y

* Han joined the Roatán Institute for Marine Sciences (RIMS) study group in 2009 as an adult male from the wild. ** Ritchie and Gracie were wild caught, all other dolphins were born at RIMS.

**Table 2:** Summary of construct operational definitions and measures.

Constructs	Fluency	Flexibility	Originality	Elaboration
*Operational Definitions*	*Correct*—number of correct different behaviors presented in a session *Percent correct*	*Energy*—high (H), moderate (M), low (L), and combinations of same or different compound energy levels	*Unique*	*Simple*
		*Type*— motor (M), vocal (V), bubbles (B), or combinations of same or different compound types	*Single*	*Sequence*
	*Repeat*—number of consecutively reinforced behaviors (trials) before a repeat.		*True original*	*Simultaneous*
			*Invented*	
Measures	*Correct*: reinforced N *Percent Correct*: Correct/total trials *Repeat*: consecutively reinforced new behaviors before a repeat (*N_R_)*	*Energy*: 1 = L 2 = M 3 = H 4 = L + L 5 = M + M 6 = H + H 7 = L + M 8 = L + H 9 = M + H 10 = L + M + H	*Unique*: performed by only 1 dolphin	*Single action*: 1 behavior performed
		*Type*: 1 = M 2 = V 3 = B 4 = M + M 5 = V + V 6 = B + B 7 = M + V 8 = M + B 9 = V + B 10 = M + V + B	*Single*: behavior emitted a single time across sessions for one dolphin (may have been produced by other dolphins	*Sequence*: behaviors performed in sequence in a trial
			*True original*: behavior emitted a single time across all sessions and all dolphins	*Simultaneous:* behaviors performed at the same time
			Invented: behavior emitted that had never been seen in a dolphin’s stimulus control repertoire	

Note. Definitions and coding process were adapted from Hill et al. [37]. Examples may be found in the text.

**Table 3:** Data from each male dolphin for Sessions 2 to 5. Abbreviations: F1 is the number of reinforced trials, F2 is fluency percent correct, F3 is total attempted trials, and F4 is the trials completed before a repeat behavior; flexibility energy and type columns 1 through 10 (defined in Table 3, Hill et al. [37]) and frequency of trials for elaboration 1 (single), 2 (sequence), and 3 (simultaneous).

		Originality				Fluency				Flexibility																						
Animal	Session #	Unique	Single	True Original	Total	F1	F2 (%)	F3	F4	Energy								Type												Elaboration		
										1	2	3	4	5	6	7	8	9	10	1	2	3	4	5	6	7	8	9	10	1	2	3
Bill	2	0	3	1	4	9	75.0%	12	4	2	0	1	2	1	1	1	1	0	0	2	0	1	6	0	0	0	0	0	0	4	5	0
	3	0	8	6	14	18	52.9%	34	5	5	4	4	3	0	0	0	2	0	0	10	2	1	3	0	0	2	0	0	0	11	7	0
	4	0	2	2	4	12	60.0%	20	2	6	1	2	0	0	0	1	1	0	1	8	2	0	1	0	0	1	0	0	0	8	4	0
	5	1	4	3	8	20	54.1%	37	2	6	3	5	2	0	0	2	1	1	0	8	4	1	4	0	0	1	2	0	0	13	6	1
French	2	0	3	5	8	15	88.2%	17	7	4	4	3	0	0	0	0	0	3	1	8	3	0	4	0	0	0	0	0	0	9	6	0
	3	0	0	0	0	5	38.5%	13	1	1	1	2	0	0	0	0	1	0	0	4	0	0	1	0	0	0	0	0	0	4	1	0
	4	0	1	4	5	10	45.5%	22	4	1	1	2	1	1	1	1	0	2	0	3	0	0	5	0	0	1	1	0	0	4	6	0
	5	1	3	11	15	18	47.4%	38	2	4	3	2	0	1	0	4	1	3	0	6	3	0	6	0	0	1	2	0	0	7	1	1
Ritchie	2	0	2	0	2	9	50.0%	18	4	2	3	1	1	0	0	0	1	1	0	6	0	0	2	0	0	1	0	0	0	6	3	0
	3	0	3	1	4	9	33.3%	27	2	2	3	2	2	0	0	0	0	0	0	7	0	0	1	0	0	1	0	0	0	7	2	0
	4	3	4	2	9	13	48.2%	27	1	2	2	3	0	0	0	3	0	3	0	6	1	0	4	0	0	1	1	0	0	7	6	0
	5	1	2	3	6	8	53.3%	15	2	2	2	1	1	0	0	1	1	0	0	4	1	0	3	0	0	0	0	0	0	5	3	0
Han	2	0	4	1	5	13	61.9%	21	2	3	4	3	0	1	0	2	0	0	0	9	0	1	1	0	0	0	0	2	0	6	7	0
	3	1	8	11	20	24	63.2%	38	6	7	3	4	4	0	0	4	0	2	0	9	3	1	5	0	0	2	4	0	0	10	12	2
	4	4	1	3	8	15	75.0%	20	3	2	3	2	1	0	1	1	1	4	0	7	0	0	6	0	0	2	0	0	0	8	7	0
	5	1	3	2	6	15	53.6%	28	1	4	1	3	2	0	0	4	1	0	0	6	2	0	3	0	0	1	3	0	0	8	7	0
Ronnie	2	0	3	6	9	11	68.8%	16	2	2	3	3	1	0	0	0	1	1	0	8	1	0	1	0	0	0	1	0	0	5	6	0
	3	1	1	6	8	19	76.9%	26	12	4	5	5	1	1	0	2	0	1	0	12	1	1	2	0	0	0	3	0	0	11	8	0
	4	1	8	7	16	20	46.5%	43	2	6	5	5	2	0	0	1	1	0	0	13	3	0	1	0	0	0	3	0	0	11	9	0
	5	1	2	4	7	12	57.1%	21	5	2	2	4	2	0	0	0	1	1	0	6	2	0	4	0	0	0	0	0	0	7	5	0
Champ	2	0	4	1	5	12	57.1%	21	8	8	3	1	0	0	0	0	0	0	0	6	5	1	0	0	0	0	0	0	0	11	1	0
	3	1	4	5	10	15	42.9%	35	6	3	3	1	6	0	0	1	1	0	0	7	1	0	3	0	0	2	2	0	0	6	9	0
	4	1	0	1	2	12	50.0%	24	6	1	3	4	1	1	0	2	0	0	0	6	0	1	1	0	0	2	2	0	0	5	4	3
	5	0	2	3	5	15	51.7%	29	4	2	2	0	3	0	0	5	2	1	0	4	0	0	6	0	0	1	4	0	0	2	12	1
Lenca	2	0	0	1	1	2	40.0%	5	2	1	1	0	0	0	0	0	0	0	0	1	1	0	0	0	0	0	0	0	0	2	0	0
	3	0	4	5	9	9	20.9%	43	3	5	1	2	0	0	0	0	0	1	0	3	5	0	1	0	0	0	0	0	0	8	1	0
	4	1	0	0	1	4	28.6%	14	2	2	1	1	0	0	0	0	0	0	0	2	2	0	0	0	0	0	0	0	0	4	0	0
	5	0	0	0	0	4.75	32.4%	18	2.25	2.5	1	0.75	0	0	0	0	0	0.5	0	1.75	2.5	0	0.5	0	0	0	0	0	0	4.25	1	0

**Table 4:** Data from each female dolphin for Sessions 2 to 5. Abbreviations: F1 is the number of reinforced trials, F2 is fluency percent correct, F3 is total attempted trials, and F4 is the trials completed before a repeat behavior; flexibility energy and type columns 1 through 10 (defined in Table 4, Hill et al. [37]) and frequency of trials for elaboration 1 (single), 2 (sequence), and 3 (simultaneous).

		Originality				Fluency				Flexibility																						
Animal	Session #	Unique	Single	True Original	Total	F1	F2	F3	F4	Energy												Type								Elaboration		
										1	2	3	4	5	6	7	8	9	10	1	2	3	4	5	6	7	8	9	10	1	2	3
Gracie	2	0	0	0	0	5	100.0%	5	5	3	0	2	0	0	0	0	0	0	0	2	2	1	0	0	0	0	0	0	0	5	0	0
	3	0	0	0	0	4	44.4%	9	2	1	1	2	0	0	0	0	0	0	0	3	1	0	0	0	0	0	0	0	0	4	0	0
	4	1	1	0	2	9	48.0%	25	6	3	1	5	0	0	0	0	0	0	0	6	3	0	0	0	0	0	0	0	0	10	2	0
	5	1	1	0	2	7	46.7%	15	5	1	5	0	0	1	0	0	0	0	0	6	0	0	0	1	0	0	0	0	0	6	1	0
Maury	2	0	1	0	1	5	55.6%	9	2	2	1	0	1	0	0	0	1	0	0	2	1	0	1	0	0	1	0	0	0	3	2	0
	3	1	3	5	9	13	48.2%	27	3	5	2	1	0	1	0	2	0	2	0	6	2	0	3	0	0	0	2	0	0	8	5	0
	4	0	4	3	7	12	40.0%	30	3	3	0	0	1	1	0	6	0	1	0	1	2	0	3	0	0	1	5	0	0	3	9	0
	5	1	3	2	6	7	43.8%	16	4	5	1	0	0	0	0	1	0	0	1	3	3	0	0	0	0	0	1	0	1	5	2	0
Bailey	2	0	1	0	1	4	18.2%	22	2	3	1	0	0	0	0	0	0	0	0	2	2	0	0	0	0	0	0	0	0	4	0	0
	3	0	0	4	4	3	9.7%	31	2	1	1	0	0	1	0	0	0	0	0	2	0	0	1	0	0	0	0	0	0	2	1	0
	4	0	1	1	2	7	58.3%	12	5	1	5	0	0	1	0	0	0	0	0	4	2	0	1	0	0	0	0	0	0	7	1	0
	5	0	1	0	1	8	57.1%	14	5	1	2	1	2	1	0	1	0	0	0	3	1	0	1	0	0	2	1	0	0	4	3	1
Tilly	2	0	1	2	3	5	35.7%	14	4	4	1	0	0	0	0	0	0	0	0	2	3	0	0	0	0	0	0	0	0	5	0	0
	3	0	1	0	1	2	25.0%	8	1	1	0	0	0	1	0	0	0	0	0	0	1	0	1	0	0	0	0	0	0	1	1	0
	4	2	4	1	7	7	70.0%	10	6	2	4	0	1	0	0	0	0	0	0	2	1	2	1	0	1	0	0	0	0	5	1	1
	5	0	0	0	0	1	8.3%	12	1	0	1	0	0	0	0	0	0	0	0	1	0	0	0	0	0	0	0	0	0	1	0	0
Poli	2	0	2	1	3	4	44.4%	9	2	2	0	0	0	0	0	1	1	0	0	1	1	0	2	0	0	0	0	0	0	2	2	0
	3	1	3	1	5	10	43.5%	23	4	5	2	1	2	0	0	0	0	0	0	6	2	0	2	0	0	0	0	0	0	8	4	0
	4	1	1	2	4	5	41.7%	12	2	2	1	0	1	0	0	0	0	1	0	2	1	0	2	0	0	0	0	0	0	3	2	0
	5	1	2	3	6	4	28.6%	14	3	3	0	0	0	0	0	0	1	0	0	2	1	0	1	0	0	0	0	0	0	3	2	0

**Table 5:** Bottlenose dolphin rankings for three constructs.

Fluency			Flexibility			Originality		
Ranking	Dolphin	Sum z-Scores	Ranking	Dolphin	Sum z-Scores	Ranking	Dolphin	Sum z-Scores
1st	Champ	4.2608	1st	Han	2.5334	1st	Han	4.7476
2nd	Ronnie	4.2186	2nd	French	1.6584	2nd	Ronnie	3.3314
3rd	Bill	3.4854	3rd	Champ	1.5830	3rd	Bill	1.1134
4th	Han	3.1532	4th	Maury	1.5713	4th	Ritchie	0.9176
5th	French	0.5512	5th	Bill	1.5628	5th	French	0.2435
6th	Maury	0.3063	6th	Ritchie	−0.1823	6th	Maury	0.2421
7th	Gracie	−1.3096	7th	Ronnie	−0.2818	7th	Champ	0.0406
8th	Ritchie	−1.8720	8th	Bailey	−0.7510	8th	Poli	−0.1678
9th	Poli	−2.2056	9th	Tilly	−0.8508	9th	Tilly	−1.7673
10th	Bailey	−3.0629	10th	Poli	−1.8686	10th	Lenca	−2.3649
11th	Lenca	−3.1954	11th	Gracie	−2.3190	11th	Gracie	−3.0027
12th	Tilly	−4.3299	12th	Lenca	−2.6555	12th	Bailey	−3.3336

Fluency sum z-scores include fluency 1, 2, 3, and 4 z-scores. Flexibility sum z-scores include energy score and type score z-scores. Originality sum z-scores include unique, single, and true original z-scores. Elaboration was excluded due to limitations associated with behavioral criteria.

**Table 6:** Overall creativity rankings for all dolphins tested.

Ranking	Dolphin	Summed z-Scores
1st	Han	3.6375
2nd	Ronnie	2.0242
3rd	Bill	2.0239
4th	Champ	1.8703
5th	French	1.0482
6th	Maury	0.9429
7th	Ritchie	−0.2532
8th	Poli	−1.5416
9th	Tilly	−2.0970
10th	Bailey	−2.2524
11th	Gracie	−2.4878
12th	Lenca	−2.9149

Final scores used weighted overall fluency, flexibility, and originality sum z-scores.

## Data Availability

Data are available upon request.

## Supplementary Materials

The following supporting information can be downloaded at: https://www.mdpi.com/article/10.3390/ani14060896/s1, Figure S1: Percent correct per session per dolphin; Figure S2: Number of trials correct before repeat per session; Figure S3: Mean Proportion of Behaviors Emitted per Flexibility Energy Category Per Dolphin; Figure S4: Mean Proportion of Behaviors Emitted per Flexibility Type Category Per Dolphin; Figure S5: Mean Number of Behaviors Emitted per Originality Category Per Dolphin; Figure S6: Mean Proportion of Behaviors Emitted per Elaboration Category Per Dolphin.
