# Supplementary material for: Training the Concept of Innovate in Dolphins (Tursiops truncatus) Is Both Creative and Cognitively Stimulating
Source: Animals (Basel). 2024 Mar 14;14(6):896. doi: 10.3390/ani14060896 (PMC10967442; doi:10.3390/ani14060896)

Supplemental Figures for Yeater et al.

The graphical representations of each construct are provided for each dolphin in this supplemental file.

Fluency

Figure S1. Percent correct per session per dolphin.

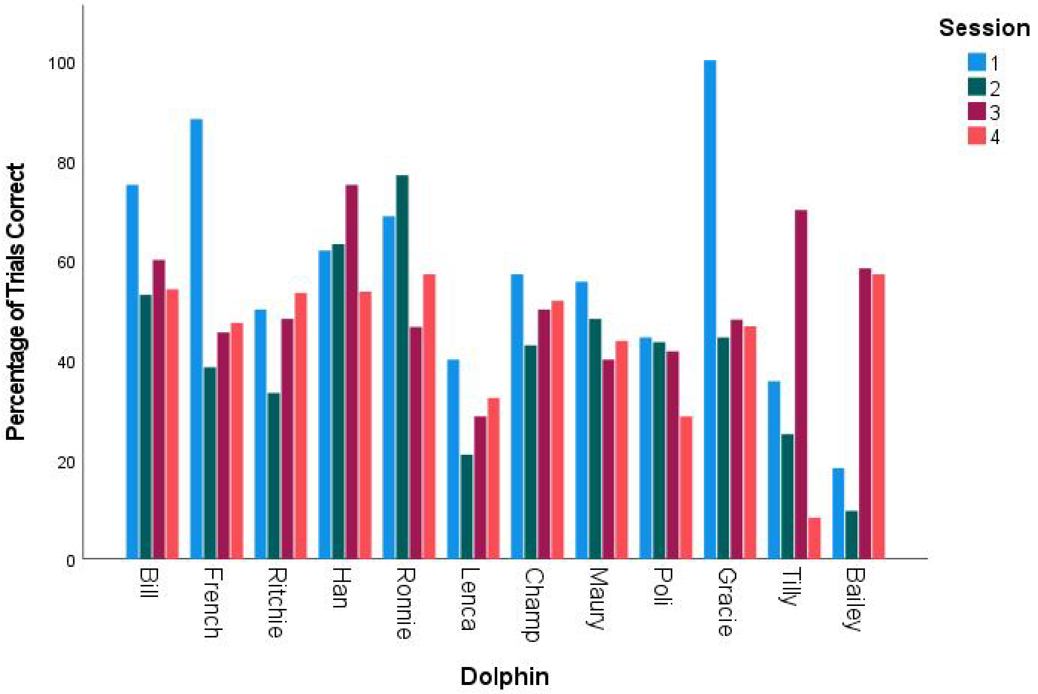

Figure S2. Number of trials correct before repeat per session.

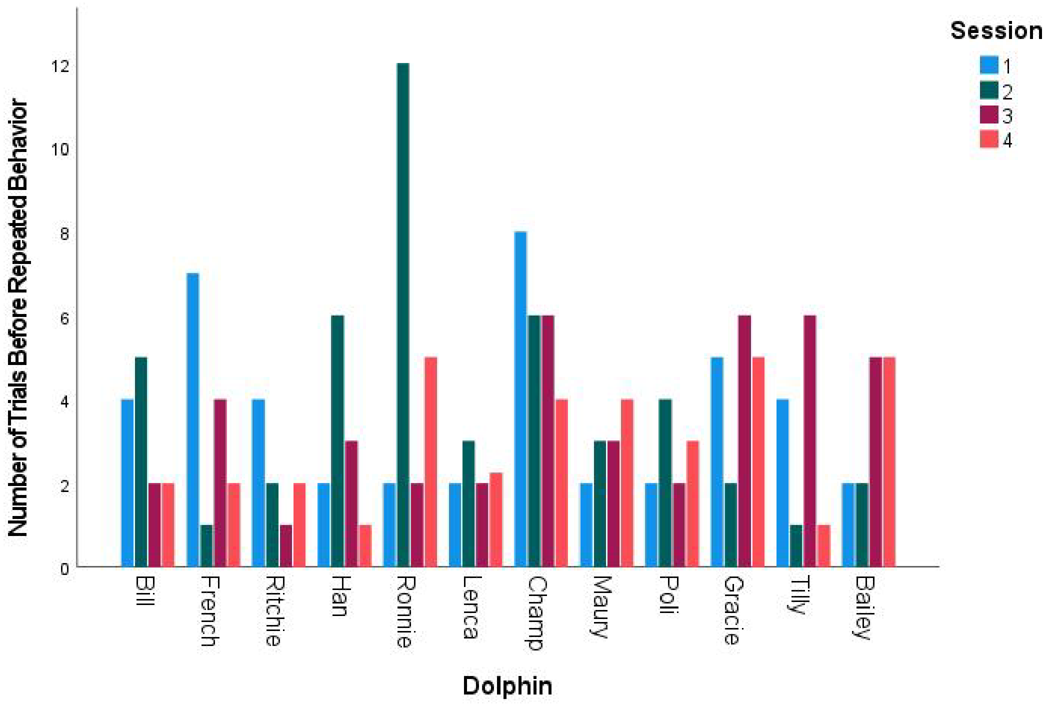

Flexibility

Figure S3. Mean Proportion of Behaviors Emitted per Flexibility Energy Category Per Dolphin.

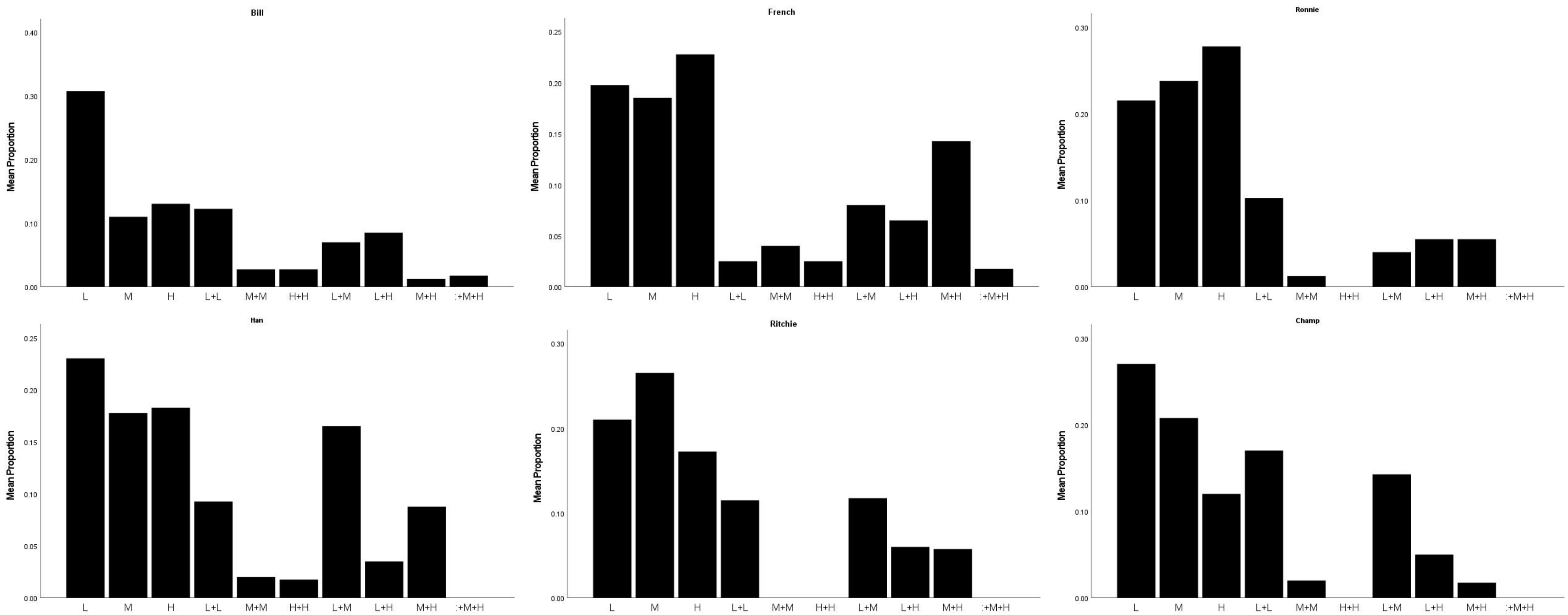

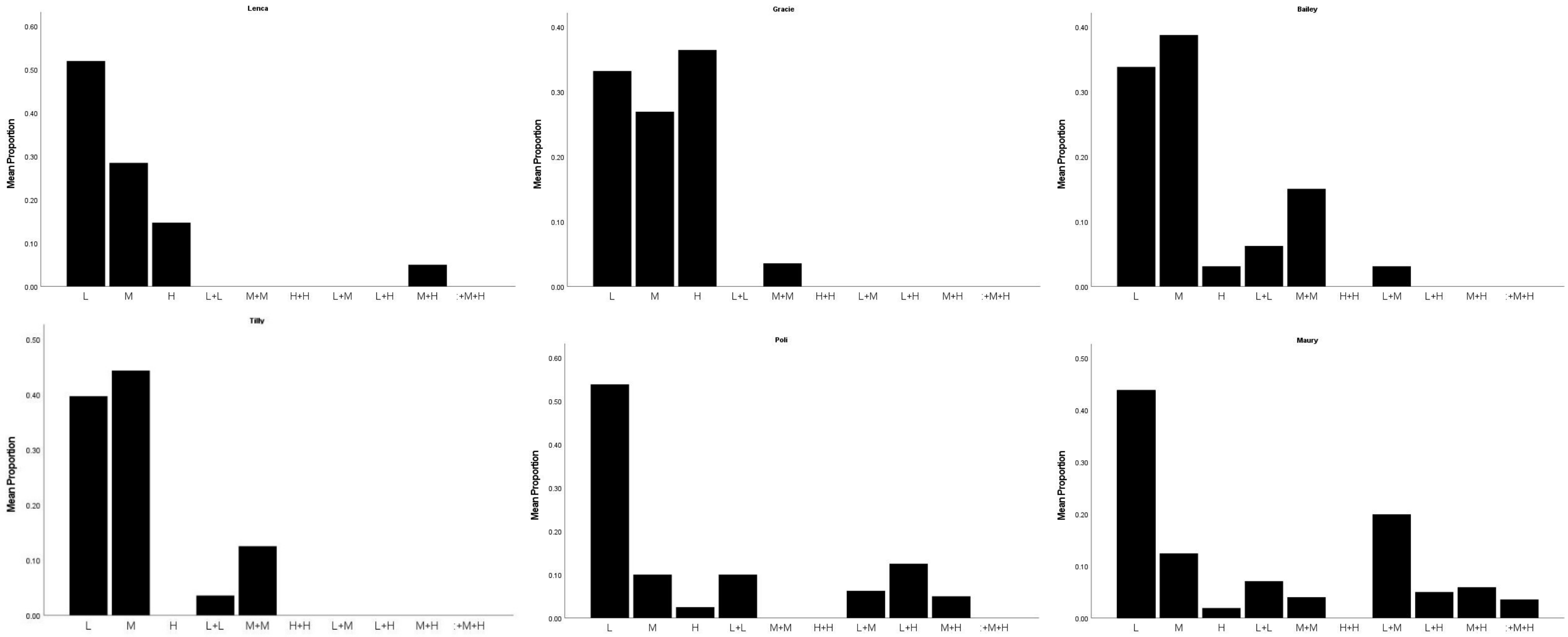

Lenca
Gracie
Bailey
Tilly
Poli
Maury
Mean Proportion
0.00
0.10
0.20
0.30
0.40
0.50
0.60
L
M
H
L+L
M+M
H+H
L+M
L+H
M+H
:+M+H

Figure S4. Mean Proportion of Behaviors Emitted per Flexibility Type Category Per Dolphin

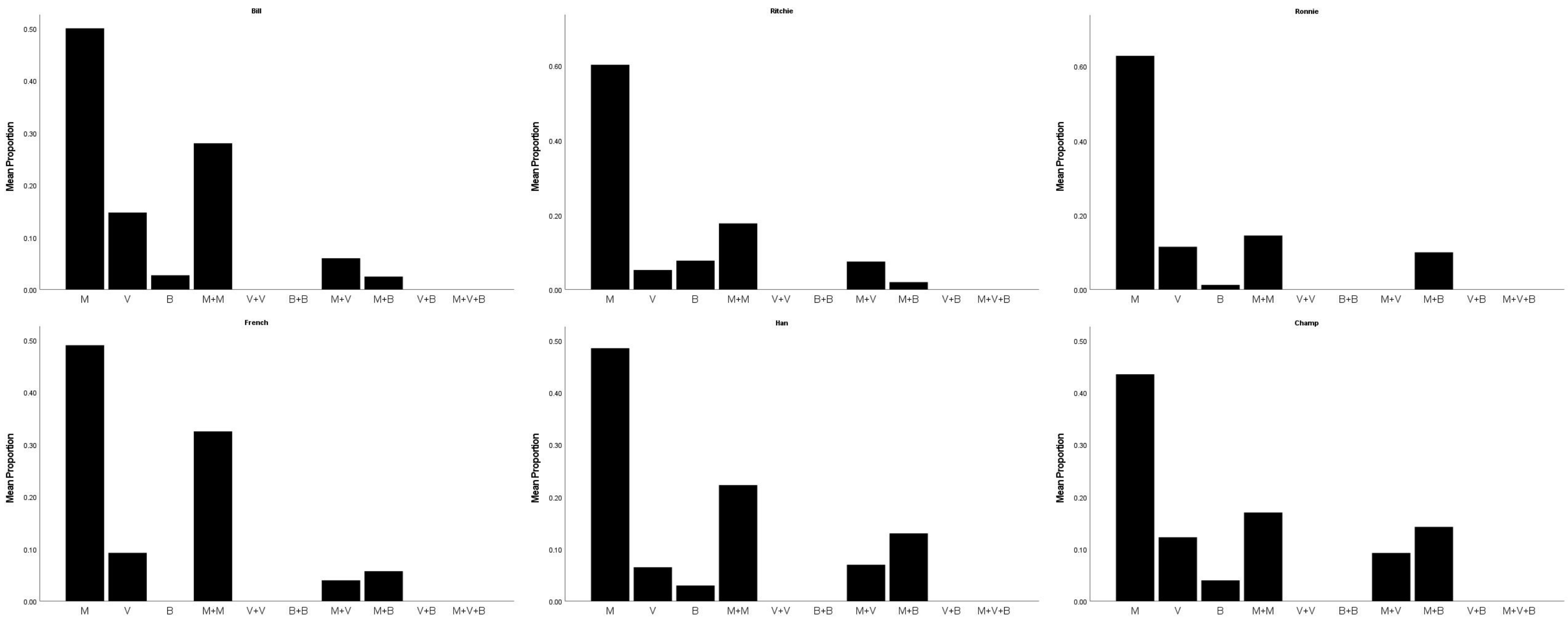

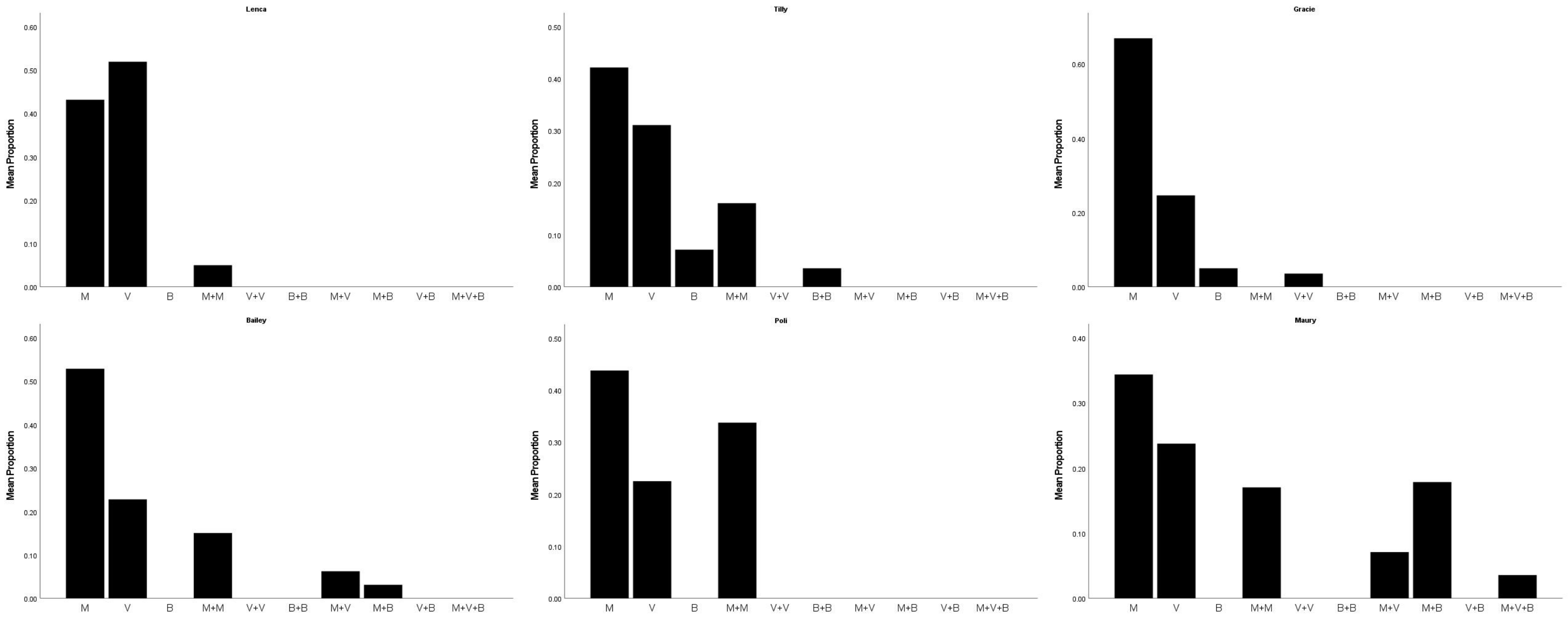
Lenca
Tilly
Gracie
Bailey
Poli
Maury
Mean Proportion
0.00
0.10
0.20
0.30
0.40
0.50
0.60
M
V
B
M+M
V+V
B+B
M+V
M+B
V+B
M+V+B

Originality

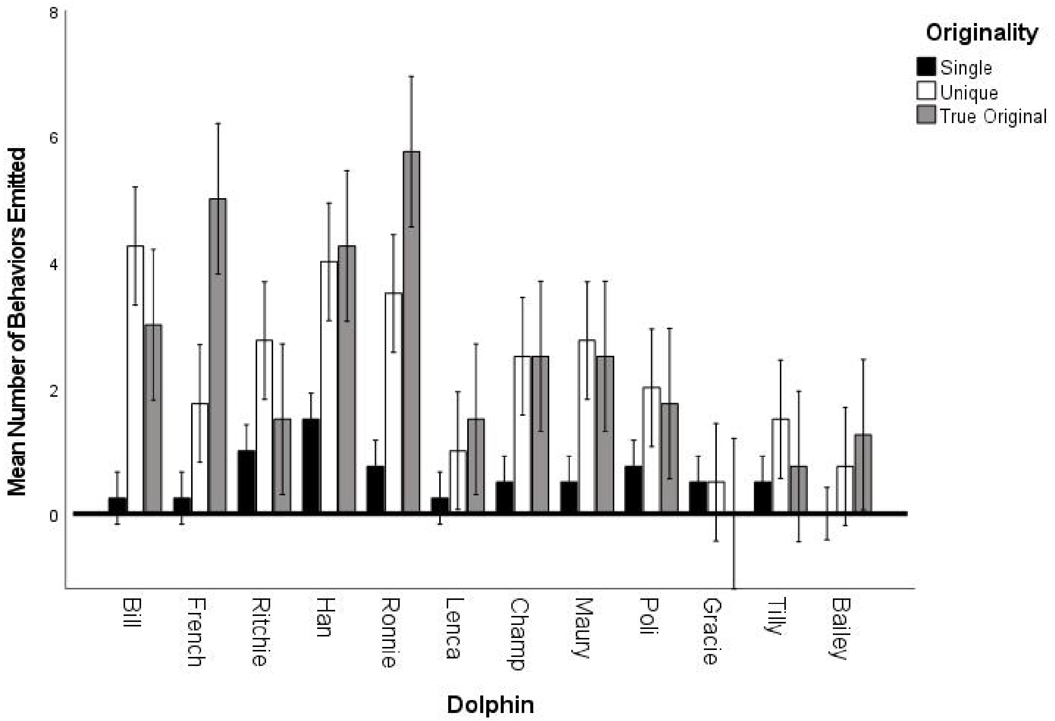

Figure S5. Mean Number of Behaviors Emitted per Originality Category Per Dolphin.

Elaboration

Figure S6. Mean Proportion of Behaviors Emitted per Elaboration Category Per Dolphin.

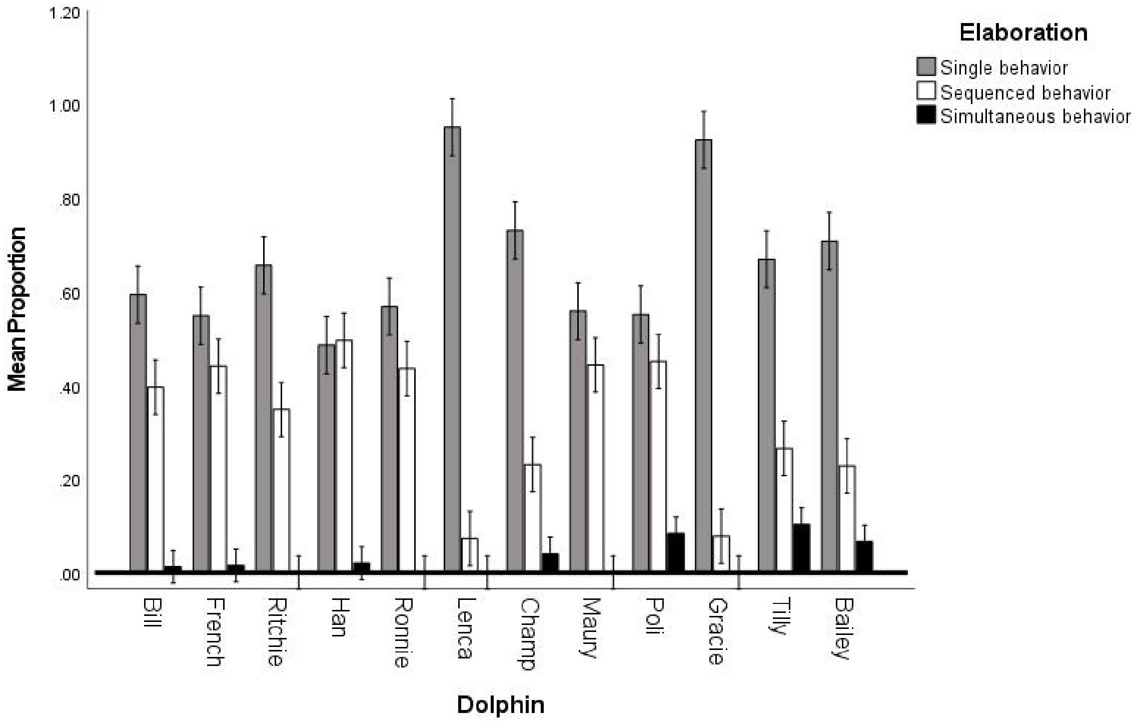

Supplement: Supplementary file 1 [file animals-14-00896-s001.zip › animals-2806643-supplementary.pdf]
